# Effect of Cheese Intake on Cardiovascular Diseases and Cardiovascular Biomarkers

**DOI:** 10.3390/nu14142936

**Published:** 2022-07-18

**Authors:** Meng-Jin Hu, Jiang-Shan Tan, Xiao-Jin Gao, Jin-Gang Yang, Yue-Jin Yang

**Affiliations:** State Key Laboratory of Cardiovascular Disease, Fuwai Hospital, National Center for Cardiovascular Diseases, Chinese Academy of Medical Sciences & Peking Union Medical College, Beijing 100037, China; 18393911603@163.com (M.-J.H.); happyshown@163.com (J.-S.T.); sophie_gao@sina.com (X.-J.G.); jingangyang@126.com (J.-G.Y.)

**Keywords:** cheese intake, cardiovascular diseases, biomarkers, Mendelian randomization, causal association

## Abstract

Background: A growing number of cohort studies revealed an inverse association between cheese intake and cardiovascular diseases, yet the causal relationship is unclear. Objective: To assess the causal relationship between cheese intake, and cardiovascular diseases and cardiovascular biomarkers. Methods: A two-sample Mendelian randomization (MR) analysis based on publicly available genome-wide association studies was employed to infer the causal relationship. The effect estimates were calculated using the random-effects inverse-variance-weighted method. Results: Cheese intake per standard deviation increase causally reduced the risks of type 2 diabetes (odds ratio (OR) = 0.46; 95% confidence interval (CI), 0.34–0.63; *p* = 1.02 × 10^−6^), heart failure (OR = 0.62; 95% CI, 0.49–0.79; *p* = 0.0001), coronary heart disease (OR = 0.65; 95% CI, 0.53–0.79; *p* = 2.01 × 10^−5^), hypertension (OR = 0.67; 95% CI, 0.53–0.84; *p* = 0.001), and ischemic stroke (OR = 0.76; 95% CI, 0.63–0.91; *p* = 0.003). Suggestive evidence of an inverse association between cheese intake and peripheral artery disease was also observed. No associations were observed for atrial fibrillation, cardiac death, pulmonary embolism, or transient ischemic attack. The better prognosis associated with cheese intake may be explained by lower body mass index (BMI; effect estimate = −0.58; 95% CI, from −0.88 to −0.27; *p* = 0.0002), waist circumference (effect estimate = −0.49; 95% CI, from −0.76 to −0.23; *p* = 0.0003), triglycerides (effect estimate = −0.33; 95% CI, from −0.50 to −0.17; *p* = 4.91 × 10^−5^), and fasting glucose (effect estimate = −0.20; 95% CI, from −0.33 to −0.07; *p* = 0.0003). There was suggestive evidence of a positive association between cheese intake and high-density lipoprotein. No influences were observed for blood pressure or inflammation biomarkers. Conclusions: This two-sample MR analysis found causally inverse associations between cheese intake and type 2 diabetes, heart failure, coronary heart disease, hypertension, and ischemic stroke.

## 1. Introduction

Dairy fat is characterized by a rich content of saturated fatty acids, which are known to elevate the level of low-density lipoprotein (LDL) cholesterol, thereby the risk of cardiovascular diseases [1]. Correspondingly, the dietary guidelines from both the American Heart Association and the European Society of Cardiology recommend lowering the intake of saturated fat, and only low-fat dairy products are suggested [2,3]. Surprisingly, a meta-analysis including 12 prospective cohort studies demonstrated no significant increases in cardiovascular diseases for a high intake of saturated fat compared with a low intake of it [4]. Another meta-analysis including 21 prospective studies also found no significant associations between dietary saturated fat intake and the risks of coronary heart disease, stroke, or cardiovascular diseases [5]. In addition, specific dairy foods may play different roles in the development of cardiovascular diseases. For example, the extensive European Investigation into Cancer and Nutrition (EPIC) cohort involving 340,234 participants across eight European countries revealed that milk intake had no associations with type 2 diabetes, while cheese intake was inversely associated with the risk of diabetes (relative ratio (RR) = 0.83; 95% CI, 0.70–0.98; *p* = 0.003) [6]. Similarly, other observational studies also found no or even inverse correlation between cheese intake and cardiovascular diseases, although cheese is usually regarded as a full-fat dairy product [7,8,9]. However, as these are observational studies in nature, a selection bias may exist, which requires us to interpret the results with caution. Regrettably, no randomized controlled trials with hard endpoints are available on this topic to prove the causality. Such a randomized controlled trial is also highly challenging to be performed in the future. However, considering the fact that dairy accounts for 10% of daily calories in the USA, and cheese takes up 45% of dairy [10], it is worthwhile to investigate the causal relationship between cheese intake and cardiovascular outcomes.

Currently, the Mendelian randomization (MR) analysis has been widely applied to assess the potential causal relationships between various exposures and clinical outcomes. Compared with traditional observational studies, the MR analysis can overcome reverse causation bias, since allelic randomization always precedes the onset of disease. Moreover, random segregation and the independent assortment of genetic polymorphisms at conception enables the MR analysis to minimize the effect of confounding factors by introducing genetic markers as instrumental variables (IVs) of exposures [11,12]. The availability of large-scale genome-wide association studies (GWASs) further enables the exploration of causality. Therefore, by applying an MR analysis, we are determined to answer the following two key questions: (1) is cheese intake negatively, neutrally, or positively associated with cardiovascular diseases? (2) what is the effect of cheese intake on cardiovascular-related biomarkers?

## 2. Materials and Methods

### 2.1. Study Design

**Figure 1:**
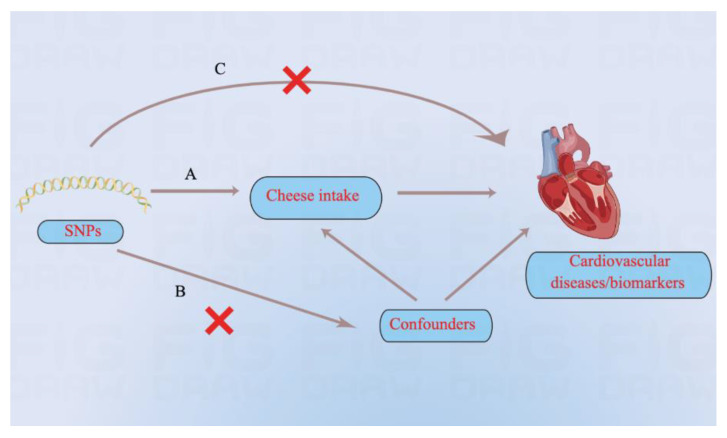
Three key assumptions of the Mendelian randomization study. (A) SNPs are strongly associated with cheese intake; (B) SNPs are independent of confounders; (C) SNPs must only affect cardiovascular diseases and biomarkers via cheese intake. SNP: single-nucleotide polymorphism.

The schematic view of the study design, and the three key assumptions of MR are shown in Figure 1 and are as follows: (A) single nucleotide polymorphisms (SNPs) are strongly associated with cheese intake; (B) SNPs are independent of known confounders; (C) SNPs only affect cardiovascular diseases and biomarkers via cheese intake (Figure 1) [13].

### 2.2. Data Sources

The analysis was conducted using published summary-level data from GWASs of the traits of interest in predominantly European individuals and included both males and females. GWAS summary statistics for cheese intake (*n* = 451,486) were obtained from the UK biobank study, which assessed the relationship between the quantity of cheese intake and SNPs [14]. Coronary heart disease (*n* = 184,305) was obtained from CARDIoGRAMplusC4D Consortium [15]. Hypertension (*n* = 218,792), atrial fibrillation (*n* = 127,442), cardiac death (*n* = 218,792), pulmonary embolism (*n* = 218,792), transient ischemic attack (*n* = 214,634), and peripheral artery disease (*n* = 218,792) were obtained from FinnGen Consortium. Heart failure (*n* = 977,323) was obtained from the Heart Failure Molecular Epidemiology for Therapeutic Targets consortium (HERMES) [16]. Type 2 diabetes (*n* = 149,821) was obtained from the results reported by Morris et al. [17]. Ischemic stroke (*n* = 440,328) was obtained from the results reported by Malik, R., et al. [18]. For cardiovascular biomarkers, systolic blood pressure (SBP, *n* = 757,601) and diastolic blood pressure (DBP, *n* = 757,601) were obtained from International Consortium of Blood Pressure (ICBP). Body mass index (BMI, *n* = 339,224) and waist circumference were obtained from the Genetic Investigation of ANthropometric Traits consortium (GIANT). C-reactive protein (CRP, *n* = 204,402) was obtained from the data reported by Ligthart et al. [19]. Interleukin 6 (*n*= 3,394) was analyzed by Folkersen et al. [20]. Adiponectin (*n*= 39,883) was analyzed by Dastani et al. [21]. Total cholesterol (*n*= 187,365), triglycerides (*n*= 177,861), high-density lipoprotein (HDL, *n* = 187,167), and LDL (*n* = 173,082) were obtained from Global Lipids Genetics Consortium (GLGC). Fasting glucose (*n* = 58,074) was obtained from Meta-Analyses of Glucose and Insulin-related traits Consortium (MAGIC). Ethics approval was not required for the current analysis as all included GWAS data are publicly available and had been approved by the corresponding ethical review boards.

### 2.3. Selection and Validation of SNPs

Three criteria were applied to select suitable SNPs. First, we selected SNPs associated with cheese intake at the genome-wide significance threshold with *p* < 5 × 10^−8^. Second, the independence among the selected SNPs was evaluated according to the pairwise-linkage disequilibrium [22]. When r^2^ > 0.001 (clumping window of 10,000 kb), the SNP correlated with more SNPs or with a higher *p*-value was deleted. Third, the F-statistic was calculated to validate the strength of individual SNPs. When F-statistics were greater than ten, SNPs were considered powerful enough to mitigate the influence of potential bias. Before performing the MR analysis, we also conducted data-harmonization steps, as the effects of an SNP on the exposure and the outcome had to correspond to the same allele.

### 2.4. MR Analysis

An inverse-variance weighted (IVW) meta-analysis under a random-effects model was regarded as the primary analysis. The following two methods, including weighted median and MR-Egger, were performed as sensitivity analyses. The weighted-median method can provide valid estimates if more than 50% of information comes from valid IVs [23]. The MR-Egger method can be used to assess the horizontal pleiotropy of selected IVs [24]. Cochrane’s Q-value can indicate heterogeneity among selected IVs. Additionally, a leave-one-out sensitivity analysis was conducted to determine whether the overall estimates were disproportionately affected by an individual SNP. To account for multiple testing in cheese intake with cardiovascular outcomes and biomarkers, Bonferroni-corrected thresholds of *p* < 0.005 (α = 0.05/10 outcomes) and *p* < 0.0042 (α = 0.05/12 biomarkers) were used for cardiovascular outcomes and biomarkers, respectively. When the *p*-value was between the Bonferroni-corrected value and 0.05, suggestive evidence of association was considered, and further confirmation was required. All statistical analyses were performed using the “TwoSampleMR” packages in R version 4.0.3 (R Foundation for Statistical Computing, Vienna, Austria).

## 3. Results

### 3.1. SNP Selection and Validation

In summary, the included studies were published between 2012 and 2021 and were mainly based on the European population (Supplementary Table S1). Sixty-five IVs achieved genome-wide significance levels, and all F-statistics were greater than ten (Supplementary Table S2).

### 3.2. Cardiovascular Diseases

**Figure 2:**
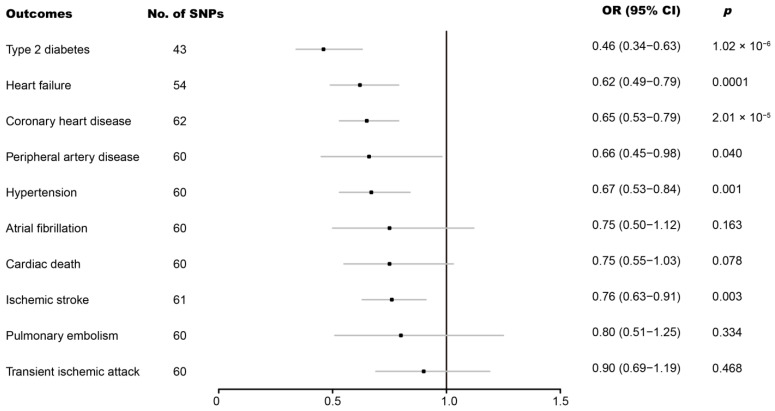
Associations of genetically predicted cheese intake with cardiovascular diseases. CI, confidence interval; OR, odds ratio; SNP, single-nucleotide polymorphism.

The IVW analysis revealed that the genetically predicted cheese intake per standard-deviation increase was inversely associated with five of the ten cardiovascular diseases, including, with decreasing magnitude of association, type 2 diabetes (odds ratio (OR) = 0.46; 95% confidence interval (CI), 0.34–0.63; *p* = 1.02 × 10^−6^), heart failure (OR = 0.62; 95% CI, 0.49–0.79; *p* = 0.0001), coronary heart disease (OR = 0.65; 95% CI, 0.53–0.79; *p* = 2.01 × 10^−5^), hypertension (OR = 0.67; 95% CI, 0.53–0.84; *p* = 0.001), and ischemic stroke (OR = 0.76; 95% CI, 0.63–0.91; *p* = 0.003) (Figure 2). Suggestive evidence of an inverse association between genetically predicted cheese intake and peripheral artery disease was also observed. In contrast, no associations were observed for atrial fibrillation, cardiac death, pulmonary embolism, or transient ischemic attack (Figure 2). For most cardiovascular diseases, the weighted-median and MR-Egger analyses revealed consistent estimates but of low precision (Table 1). No evidence of directional pleiotropy was detected. The heterogeneity was higher for some cardiovascular diseases. Therefore, an IVW meta-analysis under a random-effects model was adopted to mitigate the influence of heterogeneity.

Scatter plot and forest plot of the association between cheese intake and cardiovascular diseases are shown in Supplementary Figure S1 and Supplementary Figure S2, respectively, where similar results can be observed. The leave-one-out sensitivity analysis, as shown in Supplementary Figure S3, revealed that the overall estimates were not disproportionately affected by any individual SNP. The funnel plot in Supplementary Figure S4 also indicated no evidence of horizontal pleiotropy.

### 3.3. Cardiovascular Biomarkers

**Figure 3:**
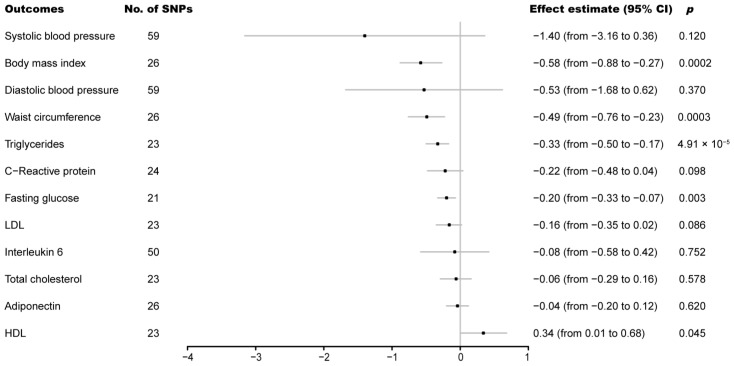
Associations of genetically predicted cheese intake with cardiovascular biomarkers. CI, confidence interval; HDL, high-density lipoprotein; LDL, low-density lipoprotein; SNP, single-nucleotide polymorphism.

The IVW analysis showed that genetically predicted cheese intake per standard-deviation increase was inversely associated with 4 of the 12 cardiovascular biomarkers, including, with decreasing magnitude of association, BMI (effect estimate = −0.58; 95% CI, from −0.88 to −0.27; *p* = 0.0002), waist circumference (effect estimate = −0.49; 95% CI, from −0.76 to −0.23; *p* = 0.0003), triglycerides (effect estimate = −0.33; 95% CI, from −0.50 to −0.17; *p* = 4.91 × 10^−5^), and fasting glucose (effect estimate = −0.20; 95% CI, from −0.33 to −0.07; *p* = 0.0003) (Figure 3). Suggestive evidence of a positive association between genetically predicted cheese intake and HDL was also observed. No associations were observed for SBP, DBP, CRP, LDL, Interleukin 6, total cholesterol, or adiponectin (Figure 3). The weighted-median and MR-Egger analyses revealed similar estimates but of low precision. No evidence of directional pleiotropy was detected in the majority of biomarkers except for HDL (Table 2).

Scatter plot, forest plot, the results of the leave-one-out sensitivity analysis, and the funnel plot of the association between cheese intake and cardiovascular biomarkers are shown in Supplementary Figure S5, Supplementary Figure S6, Supplementary Figure S7, and Supplementary Figure S8, respectively, where similar results can be observed.

## 4. Discussion

In the present two-sample MR analysis, we comprehensively assessed the causal association between cheese intake and cardiovascular diseases as well as cardiovascular biomarkers. Our results revealed that cheese intake was inversely associated with type 2 diabetes, heart failure, coronary heart disease, hypertension, and ischemic stroke, which may be explained by decreased BMI, waist circumference, triglycerides, and fasting glucose.

As a full-fat dairy product, cheese may be intuitively associated with high risks of cardiovascular diseases due to high content of saturated fatty acids and its effect on blood cholesterol [1]. However, recent studies showed that the scenario may not be the truth. On one hand, meta-analyses failed to reveal significant associations between a high intake of saturated fatty acids and cardiovascular diseases [4,5]. On the other hand, the effect of food on a single biomarker such as blood cholesterol may be insufficient to assess the risk of cardiovascular disease [25]. In that case, it may be inappropriate to assess the effect of cheese on cardiovascular disease just based on saturated fatty acids. Therefore, we regarded cheese as a food matrix and applied an MR analysis to explore its association with cardiovascular diseases. Contrary to common belief, cheese intake could actually reduce the risks of type 2 diabetes, heart failure, coronary heart disease, hypertension, and ischemic stroke. Concordant with our MR results, a meta-analysis of prospective cohort studies also revealed an inverse association between cheese intake and coronary artery disease (RR = 0.82; 95% CI, 0.72–0.93) as well as stroke (RR = 0.87; 95% CI, 0.77–0.99) [26]. Moreover, the meta-analysis conducted by Gao et al. concluded that cheese intake was associated with a reduced risk of type 2 diabetes (RR = 0.82; 95% CI, 0.77–0.87) [27]. Guo et al. also revealed that cheese intake was marginally inversely related to cardiovascular diseases (RR = 0.98, 95% CI, 0.95–1.00), while no relationship was observed for mortality (RR = 0.99, 95% CI, 0.96–1.01) [28]. However, different from previous meta-analyses mainly based on observational studies, our results derived from the MR analysis may provide a more solid conclusion, as the MR analysis is not influenced by confounders or reverse causality.

The beneficial effects of cheese on cardiovascular diseases may be explained by the following mechanisms: First, in the included SNPs, rs13257887 is located in the MSRA gene, and MSRA-transgenic animals were found to be more resistant to oxidative stress [29]. rs62034322 is located in the IL27 gene, which was able to limit chronic inflammatory pathology [30]. rs1291145 is located in the SAMHD1 gene, which also played a significant role in immune and inflammation [31]. It is well-known that oxidative stress and inflammation have significant effects on the development and progression of cardiovascular diseases [32]. Second, apart from saturated fatty acids, cheese is also rich in various minerals, such as calcium. Calcium has been reported to be inversely correlated with the level of total cholesterol and LDL by binding with fatty acids in the intestine to form insoluble soap, which may inhibit the absorption of fatty acids [33]. Additionally, both calcium and dairy had inverse relationships with blood pressure and hypertension [34]. Calcium may also reduce the risk of stroke [35]. Third, cheese contains a large amount of proteins, including casein, α-lactalbumin, and β-lactoglobulin and less abundant proteins, such as lactoferrin [36], which could reduce inflammation in rodent models with chronic disease [37]. Therefore, although saturated fat may increase the levels of inflammation, dairy-product proteins had neutral or even beneficial effects on inflammation [38]. Fourth, cheese belongs to probiotic foods, which contain a large amount of live microorganisms. Microorganisms can bind with the bile acids in the intestine, thereby inhibiting the reabsorption of bile acids into the enterohepatic circulation. The short-chain fatty acids produced by microorganisms in the intestine may alter cholesterol synthesis [39]. The main members of probiotic bacteria in cheese, Lactobacillus and Bifidobacterium genera [40], were demonstrated to have beneficial effects on immunity, inflammation, and cardiovascular risk factors in clinical trials [41]. A meta-analysis including 15 randomized controlled trials revealed that probiotics could reduce body weight (−0.60 kg), BMI (−0.27 kg/m^2^), and fat percentage (0.60%) in overweight and obese subjects [42]. Another meta-analysis found that probiotics could significantly improve blood glucose and insulin resistance [43]. Therefore, when administered in an adequate quantity, cheese may modulate the gut microbiome and have beneficial effects on the host [44]. Fifth, saturated fat in dairy could increase the LDL particle size, which may decrease the ability to permeate into the arterial wall [45]. Quebec Cardiovascular Study with 13-year follow-up data revealed that small dense LDL particles could increase the risk of ischemic heart disease, while that was not the case for large LDL particles [46]. Additionally, the small dense LDL particles in dairy products are present in significantly fewer quantities [47]. Cheese also contains quite a lot of conjugated linoleic acid, which was associated with lower blood pressure in rats [48] and affected the progression of atherosclerosis in rabbits [49].

Therefore, the effects of cheese on cardiovascular disease should be considered as a function of the total nutrient contents instead of simply that of a single component, such as saturated fat. After all, each food involves complex physical and nutritional components that may influence the digestion, absorption, and bioactive processes and the subsequent biological effects. However, it seems that recommendations based on saturated fat fail to consider the complex matrices and correspondingly complex effects on disease. Additionally, substantial evidence indicated that compared with individual nutrients, the food matrix had a more decisive influence on chronic disease [50]. For example, although dairy products are rich in saturated fat, they were in fact associated with lower nine-year incidences of metabolic syndrome and impaired fasting glycaemia and/or type 2 diabetes according to the D.E.S.I.R. study (Data from an Epidemiological Study on the Insulin-Resistance syndrome) [51]. It is, therefore, possible that the combined action of calcium, protein, probiotics, and short-chain fatty acids existing in the matrix of cheese leads to significant beneficial effects despite the presence of saturated-fat content.

Several limitations to this study deserve our attention. First, completely excluding the influence of potential directional pleiotropy is difficult in any MR study. However, evidence of pleiotropic effects was not observed in most MR-Egger intercept tests except for HDL, and similar results were observed in the sensitivity analyses. Second, the examined GWASs were primarily conducted in individuals of European ancestry, which might limit the generalization of our findings to other ethnicities. After all, dairy may exert different effects on individuals from European countries and other countries. A major component of dairy, lactose, cannot be metabolized by most East Asians [52]. Nonetheless, the European origin makes the population-stratification bias unlikely to influence our results. Third, there is a very large number of cheeses with very different compositions. However, the original GWAS was just focused on the population who consumed cheese with no details about whether it was being consumed with other diets. We had no details about the type or the duration of cheese intake, which limited us from conducting a further analysis.

## 5. Conclusions

This two-sample MR analysis found a causally inverse association between cheese intake and cardiovascular diseases including type 2 diabetes, heart failure, coronary heart disease, hypertension, and ischemic stroke, as well as a causally inverse association among cardiovascular biomarkers including BMI, waist circumference, triglycerides, and fasting glucose. No influences were observed for blood pressure or inflammation biomarkers. The intake of cheese may open up new opportunities for the management of cardiovascular diseases in the future.

## Figures and Tables

**Table 1:** Associations between genetically predicted cheese intake and cardioartery disease in sensitivity analyses using the weighted-median and MR-Egger methods.

Outcome	Weighted Median		MR-Egger		Pleiotropy		Heterogeneity	
	OR (95% CI)	*p*	OR (95% CI)	*p*	Intercept	*p*	Q	*p*
Coronary heart disease	0.65 (0.51–0.84)	0.001	1.14 (0.49–2.66)	0.757	−0.010	0.18	84	0.02
Hypertension	0.73 (0.55–0.96)	0.023	1.45 (0.55–3.82)	0.450	−0.014	0.11	107	<0.01
Atrial fibrillation	0.83 (0.50–1.39)	0.483	2.59 (0.47–14.15)	0.277	−0.021	0.15	80	0.04
Heart failure	0.85 (0.67–1.08)	0.172	0.85 (0.31–2.34)	0.750	−0.005	0.54	135	<0.01
Type 2 diabetes	0.67 (0.51–0.90)	0.007	1.65 (0.39–7.03)	0.50	−0.021	0.08	153	<0.01
Transient ischemic attack	0.86 (0.56–1.32)	0.487	1.06 (0.31–3.70)	0.924	−0.003	0.79	51	0.77
Ischemic stroke	0.71 (0.55–0.91)	0.008	1.17 (0.55–2.48)	0.679	−0.008	0.25	80	0.04
Pulmonary embolism	0.81 (0.44–1.48)	0.497	0.45 (0.07–3.04)	0.417	0.010	0.55	68	0.19
Peripheral artery disease	0.72 (0.43–1.20)	0.207	0.57 (0.11–3.11)	0.520	0.003	0.86	82	0.03
Cardiac death	0.74 (0.45–1.20)	0.223	1.24 (0.29–5.23)	0.772	−0.009	0.49	52	0.73

CI, confidence interval; MR, Mendelian randomization; OR, odds ratio.

**Table 2:** Associations between genetically predicted cheese intake and cardiovascular biomarkers in sensitivity analyses using the weighted-median and MR-Egger methods.

Outcome	Weighted Median		MR-Egger		Pleiotropy		Heterogeneity	
	Effect Estimate (95% CI)	*p*	Effect Estimate (95% CI)	*p*	Intercept	*p*	Q	*p*
Systolic blood pressure	−1.14 (from −2.17 to −0.11)	0.030	0.09 (from −7.17 to 7.35)	0.981	−0.026	0.68	593	<0.01
Diastolic blood pressure	−0.52 (from −1.12 to 0.09)	0.094	1.34 (from −3.37 to 6.06)	0.579	−0.032	0.43	765	<0.01
Body mass index	−0.30 (from −0.48 to −0.12)	0.001	−0.65 (from −2.20 to 0.89)	0.416	0.001	0.92	250	<0.01
Waist circumference	−0.39 (from −0.59 to −0.19)	<0.001	−0.45 (from −1.76 to 0.86)	0.508	−0.001	0.95	136	<0.01
C-Reactive protein	−0.25 (from −0.43 to −0.06)	0.009	−0.27 (from −1.58 to 1.03)	0.688	0.001	0.94	135	<0.01
Interleukin 6	−0.23 (from −1.02 to 0.55)	0.564	−0.40 (from −2.88 to 2.07)	0.750	0.006	0.79	42	0.77
Adiponectin	−0.04 (from −0.23 to 0.14)	0.639	−0.51 (from −1.33 to 0.30)	0.228	0.007	0.26	41	0.02
Total cholesterol	−0.13 (from −0.35 to 0.09)	0.245	0.82 (from −0.20 to 1.84)	0.130	−0.014	0.10	62	<0.01
Triglycerides	−0.40 (from −0.59 to −0.21)	<0.001	−0.71 (from −1.49 to 0.07)	0.087	0.006	0.34	38	0.02
HDL	0.21 (from 0 to 0.43)	0.054	2.04 (from 0.56 to 3.51)	0.013	−0.027	0.03	157	<0.01
LDL	−0.16 (from −0.37 to 0.05)	0.133	0.18 (from −0.71 to 1.08)	0.690	−0.006	0.44	40	0.01
Fasting glucose	−0.30 (from −0.46 to −0.14)	<0.001	−0.05 (from −0.68 to 0.59)	0.881	−0.002	0.64	37	0.01

CI, confidence interval; HDL, high-density lipoprotein; LDL, low-density lipoprotein; MR, Mendelian randomization.

## Data Availability

Data available in a publicly accessible repository that does not issue DOIs. Publicly available datasets were analyzed in this study. This data can be found here: [https://gwas.mrcieu.ac.uk/datasets/].

## Supplementary Materials

The following supporting information can be downloaded at: https://www.mdpi.com/article/10.3390/nu14142936/s1, Figure S1: Scatter plot of the association of cheese intake with cardiovascular diseases; Figure S2: Forest plot of the association of cheese intake with cardiovascular diseases; Figure S3: Leave-one-out sensitivity analysis of the association of cheese intake with cardiovascular diseases; Figure S4: Funnel plot of the association of individual diabetes with eight cardiovascular diseases; Figure S5: Scatter plot of the association of cheese intake with cardiovascular biomarkers; Figure S6: Forest plot of the association of cheese intake with cardiovascular biomarkers; Figure S7: Leave-one-out sensitivity analysis of the association of cheese intake with cardiovascular biomarkers; Figure S8: Funnel plot of the association of individual diabetes with eight cardiovascular biomarkers; Table S1: Baseline characteristics of cheese intake, cardiovascular diseases, and cardiovascular biomarkers; Table S2: Single nucleotide polymorphisms used as instrumental variables in the Mendelian randomization analyses of cheese intake.

## Abbreviation

BMI	body mass index
CI	confidence interval
CRP	C-reactive protein
DBP	diastolic blood pressure
GWAS	genome-wide association study
HDL	high-density lipoprotein
IV	instrumental variable
IVW	inverse-variance weighted
LDL	low-density lipoprotein
MR	Mendelian randomization
OR	odds ratio
RR	relative ratio
SBP	systolic blood pressure
SNP	single-nucleotide polymorphism
