# Supplementary material for: Effect of Cheese Intake on Cardiovascular Diseases and Cardiovascular Biomarkers
Source: Nutrients. 2022 Jul 18;14(14):2936. doi: 10.3390/nu14142936 (PMC9318947; doi:10.3390/nu14142936)
Supplement: Supplementary file 1 [file nutrients-14-02936-s001.zip › Supplementary Figure Captions.pdf]

Supplementary Figure Captions

Supplementary Figure S1. Scatter plot of the association of cheese intake with cardiovascular diseases.

A: Cardiovascular diseases; B: Coronary heart disease; C: Hypertension; D: Atrial fibrillation; E: Heart failure; F: Type 2 diabetes; G: Ischemic stroke; H: Transient ischemic attack; I: Pulmonary embolism; J: Peripheral vascular diseases; K: Cardiac death

Each black dot indicates a SNP, plotted by the estimate of SNP on individual diabetes and the estimate of SNP on the risk of cardiovascular diseases with standard error bars. The slopes of the lines correspond to causal estimates using each of the different methods.

SNP: single nucleotide polymorphism

Supplementary Figure S2. Forest plot of the association of cheese intake with cardiovascular diseases.

A: Cardiovascular diseases; B: Coronary heart disease; C: Hypertension; D: Atrial fibrillation; E: Heart failure; F: Type 2 diabetes; G: Ischemic stroke; H: Transient ischemic attack; I: Pulmonary embolism; J: Peripheral vascular diseases; K: Cardiac death

The dot and bar indicate the causal estimate of individual diabetes on risks of cardiovascular diseases.

Supplementary Figure S3. Leave-one-out sensitivity analysis of the association of cheese intake with cardiovascular diseases.

A: Cardiovascular diseases; B: Coronary heart disease; C: Hypertension; D: Atrial fibrillation; E: Heart failure; F: Type 2 diabetes; G: Ischemic stroke; H: Transient ischemic attack; I: Pulmonary embolism; J: Peripheral vascular diseases; K: Cardiac death

The dot and bar indicate the estimates and 95% confidence interval when the specific single nucleotide polymorphism is removed.

Supplementary Figure S4. Funnel plot of the association of individual diabetes with eight cardiovascular diseases.

A: Cardiovascular diseases; B: Coronary heart disease; C: Hypertension; D: Atrial fibrillation; E: Heart failure; F: Type 2 diabetes; G: Ischemic stroke; H: Transient ischemic attack; I: Pulmonary embolism; J: Peripheral vascular diseases; K: Cardiac death

Each black dot indicates a single nucleotide polymorphism.

Supplementary Figure S5. Scatter plot of the association of cheese intake with cardiovascular biomarkers.

A: Systolic blood pressure; B: Diastolic blood pressure; C: Body mass index; D: Waist circumference; E: C-Reactive protein; F: Interleukin 6; G: Adiponectin; H: Total cholesterol; I: Triglycerides; J: HDL; K: LDL; L: Fasting glucose

Each black dot indicates a SNP, plotted by the estimate of SNP on individual diabetes and the estimate of SNP on the risk of cardiovascular diseases with standard error bars. The slopes of the lines correspond to causal estimates using each of the different methods.

HDL: high-density lipoprotein; LDL: low-density lipoprotein; SNP: single nucleotide polymorphism

Supplementary Figure S6. Forest plot of the association of cheese intake with cardiovascular biomarkers.

A: Systolic blood pressure; B: Diastolic blood pressure; C: Body mass index; D: Waist circumference; E: C-Reactive protein; F: Interleukin 6; G: Adiponectin; H: Total cholesterol; I: Triglycerides; J: HDL; K: LDL; L: Fasting glucose

The dot and bar indicate the causal estimate of individual diabetes on risks of cardiovascular diseases.

HDL: high-density lipoprotein; LDL: low-density lipoprotein

Supplementary Figure S7. Leave-one-out sensitivity analysis of the association of cheese intake with cardiovascular biomarkers.

A: Systolic blood pressure; B: Diastolic blood pressure; C: Body mass index; D: Waist circumference; E: C-Reactive protein; F: Interleukin 6; G: Adiponectin; H: Total cholesterol; I: Triglycerides; J: HDL; K: LDL; L: Fasting glucose

The dot and bar indicate the estimates and 95% confidence interval when the specific single nucleotide polymorphism is removed.
HDL: high-density lipoprotein; LDL: low-density lipoprotein

Supplementary Figure S8. Funnel plot of the association of individual diabetes with eight cardiovascular biomarkers.

A: Systolic blood pressure; B: Diastolic blood pressure; C: Body mass index; D: Waist circumference; E: C-Reactive protein; F: Interleukin 6; G: Adiponectin; H: Total cholesterol; I: Triglycerides; J: HDL; K: LDL; L: Fasting glucose

Each black dot indicates a single nucleotide polymorphism.

HDL: high-density lipoprotein; LDL: low-density lipoprotein
