# Supplementary material for: Effect of Cheese Intake on Cardiovascular Diseases and Cardiovascular Biomarkers
Source: Nutrients. 2022 Jul 18;14(14):2936. doi: 10.3390/nu14142936 (PMC9318947; doi:10.3390/nu14142936)
Supplement: Supplementary file 1 [file nutrients-14-02936-s001.zip › Supplementary Table.pdf]

Supplementary Table S1. Baseline characteristics of cheese intake, cardiovascular diseases, and cardiovascular biomarkers

Trait	Year	Author	Population	Sample Size	n case	n control	n SNP
Cheese intake	2018	Ben Elsworth	European	451,486	-	-	9,851,867
Cardiovascular diseases							
Coronary heart disease	2015	Nikpay	European (~ 74%), Asian (~ 26%)	184,305	60,801	123,504	9,455,779
Hypertension	2021	-	European	218,792	42,857	175,935	16,380,466
Atrial fibrillation	2021	-	European	127,442	10,516	116,926	16,379,586
Heart failure	2020	Shah S	European	977,323	47,309	930,014	7,773,021
Type 2 diabetes	2012	Morris	European	149,821	34,840	114,981	127,904
Ischemic stroke	2018	Malik R	European	440,328	34,217	406,111	7,537,579
Transient ischemic attack	2021	-	European	214,634	8,835	205,799	16,380,437
Pulmonary embolism	2021	-	European	218,792	4,185	214,607	16,380,466
Peripheral artery disease	2021	-	European	218,792	1,037	217,755	16,380,466
Cardiac death	2021	-	European	218,792	7,563	211,229	16,380,466
Cardiovascular biomarkers							
Systolic blood pressure	2018	Evangelou, E	European	757,601	-	-	7,088,083
Diastolic blood pressure	2018	Evangelou, E	European	757,601	-	-	7,160,619
Body mass index	2015	Locke AE	European	339,224	-	-	2,555,511
Waist circumference	2015	Shungin D	European	232,101	-	-	2,565,408
C-Reactive protein	2018	Ligthart, S	European	204,402	-	-	2,414,379
Interleukin 6	2018	Folkersen L	European	3,394	-	-	5,270,646
Adiponectin	2012	Dastani Z	European	39,883	-	-	2,675,209
Total cholesterol	2013	Willer CJ	European (83.5%)	187,365	-	-	2,446,982
Triglycerides	2013	Willer CJ	European 83.5%)	177,861	-	-	2,439,433
HDL	2013	Willer CJ	European 83.5%)	187,167	-	-	2,447,442
LDL	2013	Willer CJ	European 83.5%)	173,082	-	-	2,437,752
Fasting glucose	2012	Manning AK	European	58,074	-	-	2,625,495

HDL: high-density lipoprotein; LDL: low-density lipoprotein; SNP: single nucleotide polymorphism

Supplementary Table S2. Single nucleotide polymorphisms used as instrumental variables in the Mendelian randomization analyses of cheese intake

SNP	Chr	EA	NEA	Beta	SE	Nearby gene	F
rs78876700	1	A	G	0.018	0.003	LOC107985376	30
rs531358	1	T	C	0.013	0.002	CCDC18	32
rs2802530	1	A	G	0.019	0.003	-	30
rs6685323	1	T	C	-0.013	0.002	AQP10	30
rs2339928	2	A	G	0.015	0.002	ATAD2B	37
rs12475594	2	G	A	0.016	0.003	FANCL	30
rs504675	2	T	C	0.027	0.002	LINC01833	137
rs72970243	2	A	G	0.022	0.003	-	43
rs1514755	2	G	A	0.016	0.003	-	39
rs79184944	3	A	T	0.020	0.003	-	36
rs4296548	3	G	T	0.013	0.002	TRANK1	32
rs62245792	3	A	T	-0.018	0.003	TAFA1	32
rs77742462	3	G	A	-0.047	0.008	LINC00636	33
rs2352974	3	T	C	-0.014	0.002	TRAIP	42
rs6774906	3	C	A	0.032	0.006	XXYLT1	31
rs4681981	3	A	C	-0.012	0.002	TASOR	31
rs4860341	4	C	T	0.024	0.004	-	31
rs73096946	4	C	T	-0.021	0.003	-	45
rs13107325	4	T	C	-0.029	0.004	SLC39A8	47
rs10938397	4	G	A	-0.013	0.002	-	32
rs4692708	4	C	A	0.015	0.003	LOC105377529	32

rs26579	5	C	G	-0.013	0.002	LINC00461/MEF2C-AS2	31
rs6873324	5	C	A	-0.012	0.002	GALNT10/LOC107986465	30
rs9504123	6	C	A	0.014	0.003	LOC105374894	32
rs975303	6	G	A	0.021	0.003	LOC105374958	54
rs1931805	6	C	T	0.013	0.002	KHDRBS2	32
rs113367286	7	T	C	0.015	0.002	-	37
rs34198643	7	T	C	-0.017	0.003	MAD1L1	39
rs12672200	7	A	G	-0.014	0.002	-	33
rs9649582	7	T	A	-0.015	0.002	CHCHD3	37
rs7012814	8	A	G	-0.019	0.002	-	67
rs7386207	8	T	C	-0.012	0.002	-	30
rs13257887	8	C	T	0.016	0.003	MSRA	40
rs3911016	9	G	T	0.021	0.003	LOC105376121	39
rs4503172	9	T	C	0.013	0.002	TTLL11	32
rs1806771	10	G	T	-0.022	0.004	ARID5B	30
rs73335955	10	C	T	0.028	0.005	SORCS3	31
rs10896050	11	T	G	-0.018	0.003	-	42
rs67238148	11	T	G	0.017	0.003	OR10A6	37
rs7936836	11	A	C	0.016	0.002	HSD17B12	49
rs73024305	11	C	G	0.033	0.005	SLC37A2	44
rs12786959	11	T	A	-0.016	0.003	CHORDC1	32
rs524468	12	G	A	-0.014	0.003	SLC6A13	31
rs1024853	12	G	C	-0.013	0.002	-	32
rs7298331	12	C	A	-0.013	0.002	-	33
rs12296440	12	A	G	0.019	0.003	-	40

rs61953351	12	T	G	0.015	0.003	OASL	32
rs1073242	13	A	G	0.016	0.002	-	47
rs11620149	13	C	T	-0.018	0.003	-	30
rs17115145	14	T	C	-0.013	0.002	PRKD1	32
rs35270670	15	G	A	0.016	0.003	WHAMM	37
rs4776970	15	T	A	0.015	0.002	MAP2K5	44
rs12447542	16	A	G	0.020	0.003	RBFOX1	34
rs61734410	16	T	C	0.017	0.003	CAC-1H	40
rs62034322	16	A	G	-0.014	0.002	IL27	37
rs71386942	16	A	C	0.014	0.003	PKD1L3	33
rs11649653	16	G	C	0.014	0.002	-	37
rs919109	17	C	G	0.020	0.003	HOXB6/HOXB-AS3	38
rs2854175	17	A	C	0.017	0.003	-	44
rs12951057	17	G	C	-0.021	0.003	NSF/LRRC37A2	48
rs2960578	18	G	T	0.017	0.002	NPC1	58
rs1434511	18	T	C	0.013	0.002	MIR4527HG	33
rs1291145	20	C	T	-0.02	0.002	SAMHD1	71
rs6126641	20	A	G	0.013	0.002	LOC105372666	30
rs62236533	22	A	G	0.025	0.004	-	46

Chr: chromosome; EA: effect allele; NEA: non-effect allele; SE: standard error; SNP: single-nucleotide polymorphisms.
