# Supplementary figures and images for: Effect of Cheese Intake on Cardiovascular Diseases and Cardiovascular Biomarkers
Source: Nutrients. 2022 Jul 18;14(14):2936. doi: 10.3390/nu14142936 (PMC9318947; doi:10.3390/nu14142936)

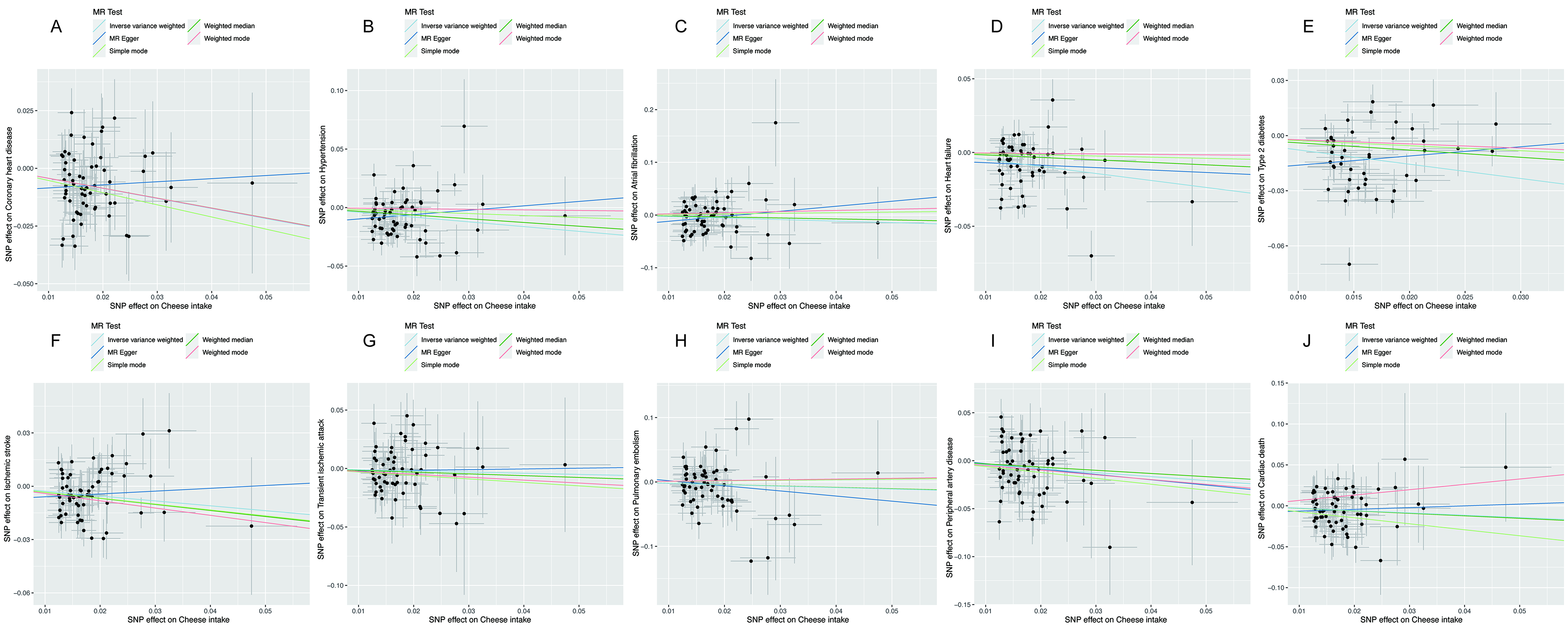

Supplement: Supplementary file 1 [file nutrients-14-02936-s001.zip › Supplementary Figure S1.tif]

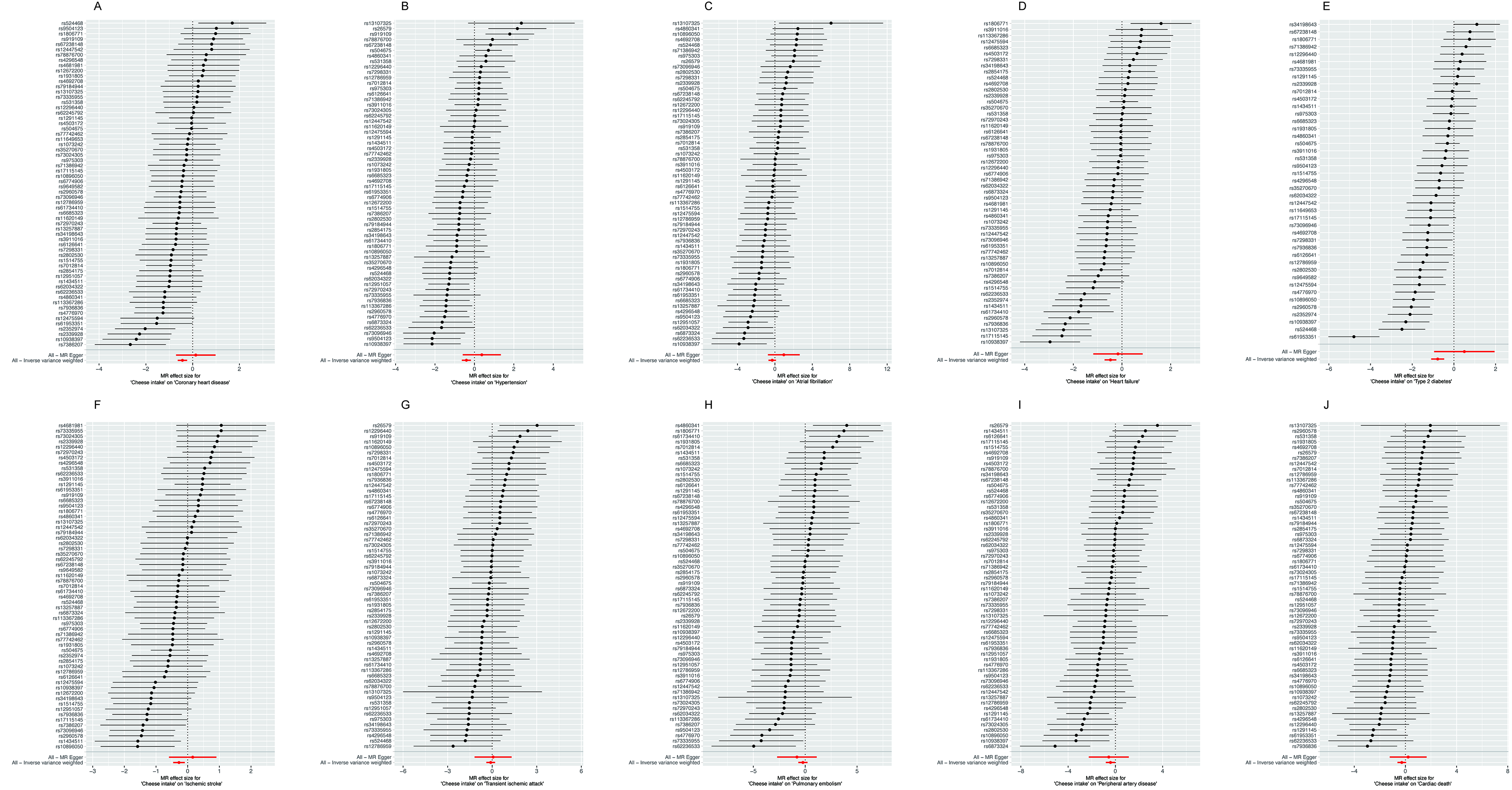

Supplement: Supplementary file 1 [file nutrients-14-02936-s001.zip › Supplementary Figure S2.tif]

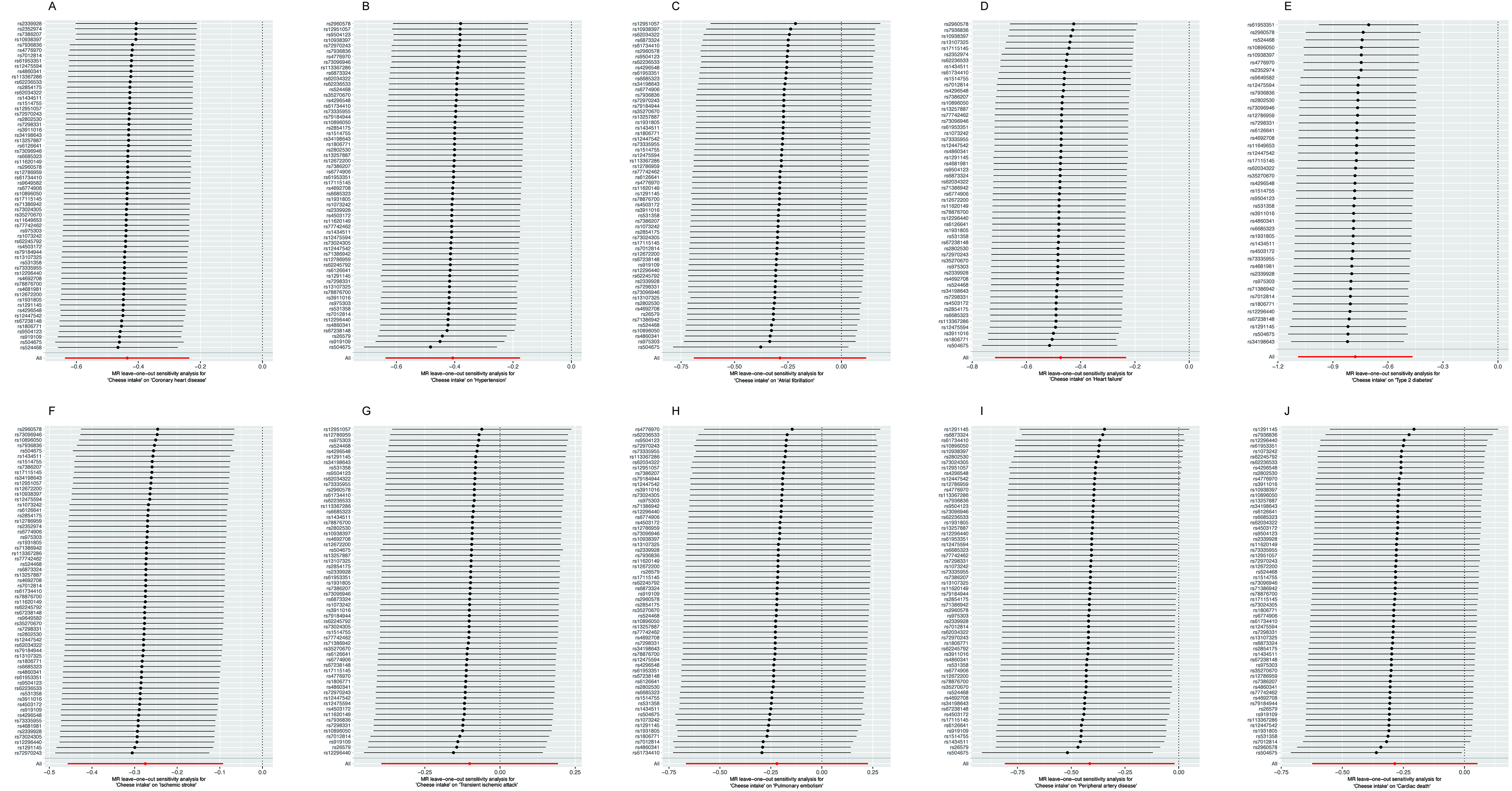

Supplement: Supplementary file 1 [file nutrients-14-02936-s001.zip › Supplementary Figure S3.tif]

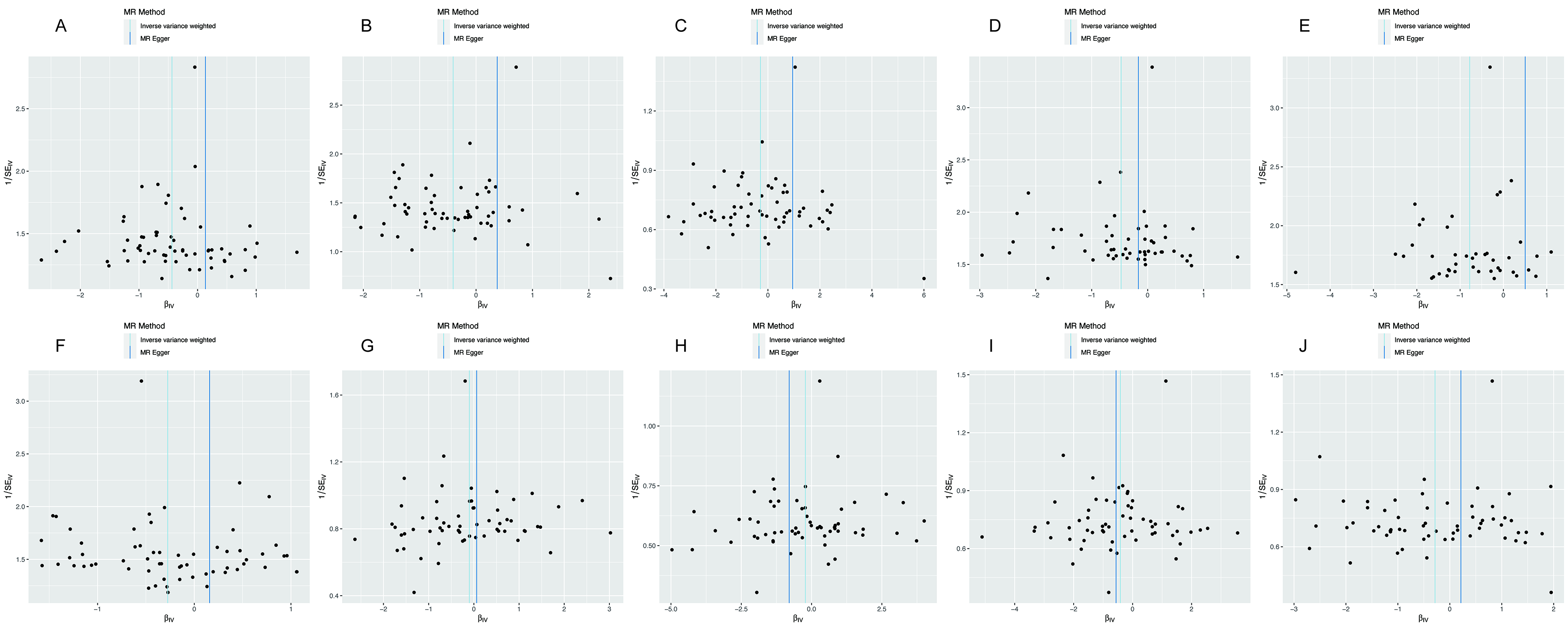

Supplement: Supplementary file 1 [file nutrients-14-02936-s001.zip › Supplementary Figure S4.tif]

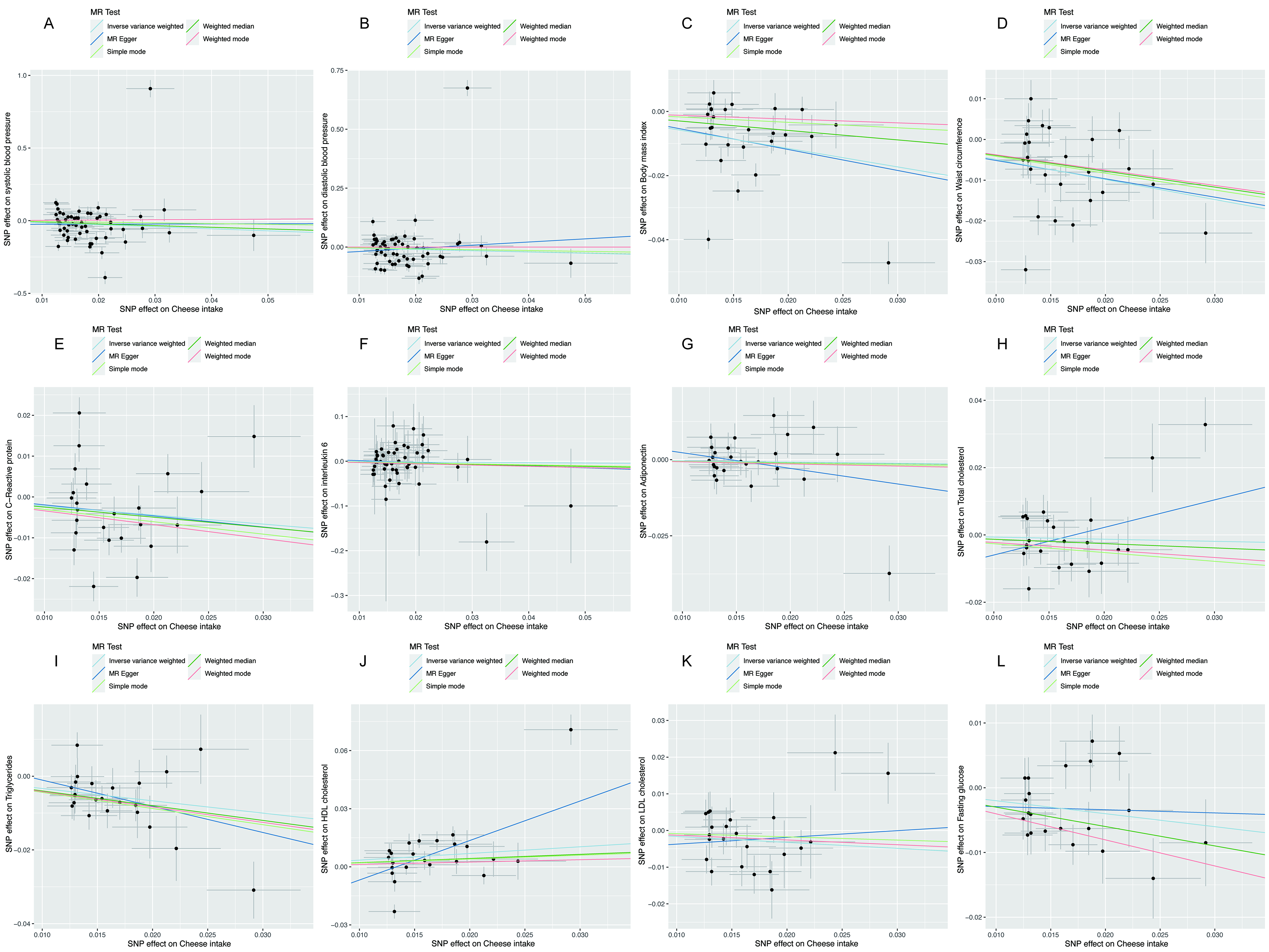

Supplement: Supplementary file 1 [file nutrients-14-02936-s001.zip › Supplementary Figure S5.tif]

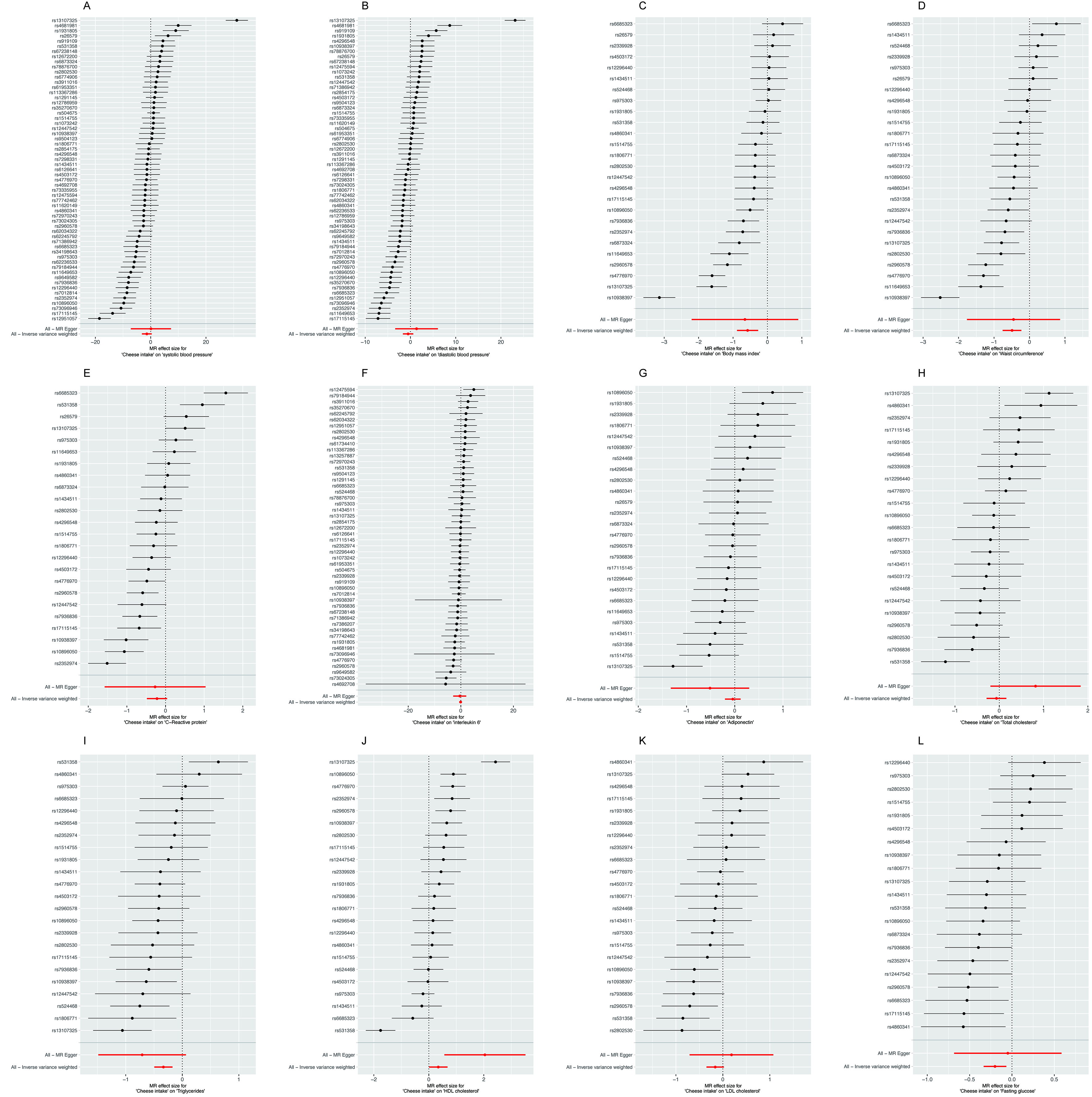

Supplement: Supplementary file 1 [file nutrients-14-02936-s001.zip › Supplementary Figure S6.tif]

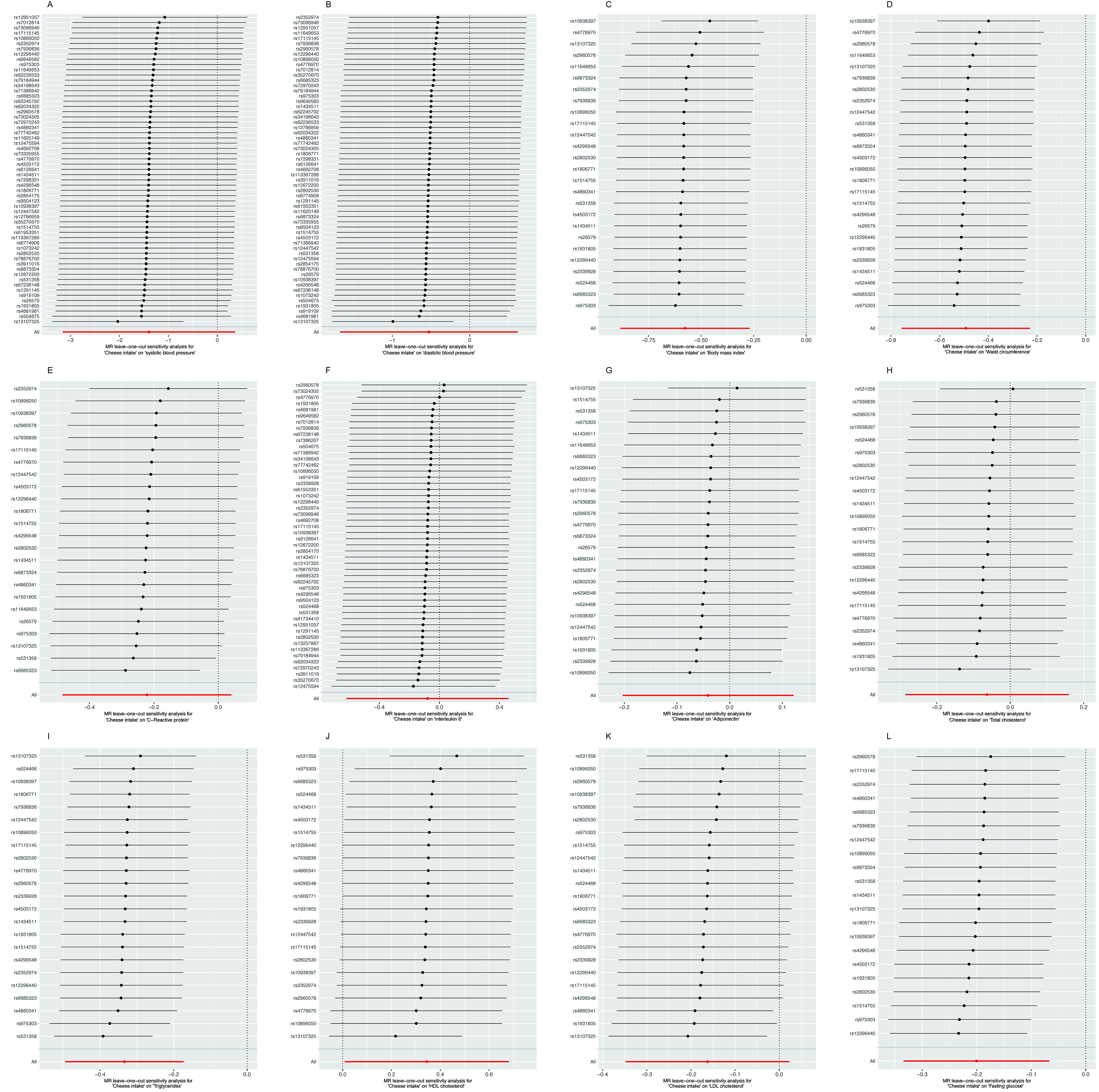

Supplement: Supplementary file 1 [file nutrients-14-02936-s001.zip › Supplementary Figure S7.tif]

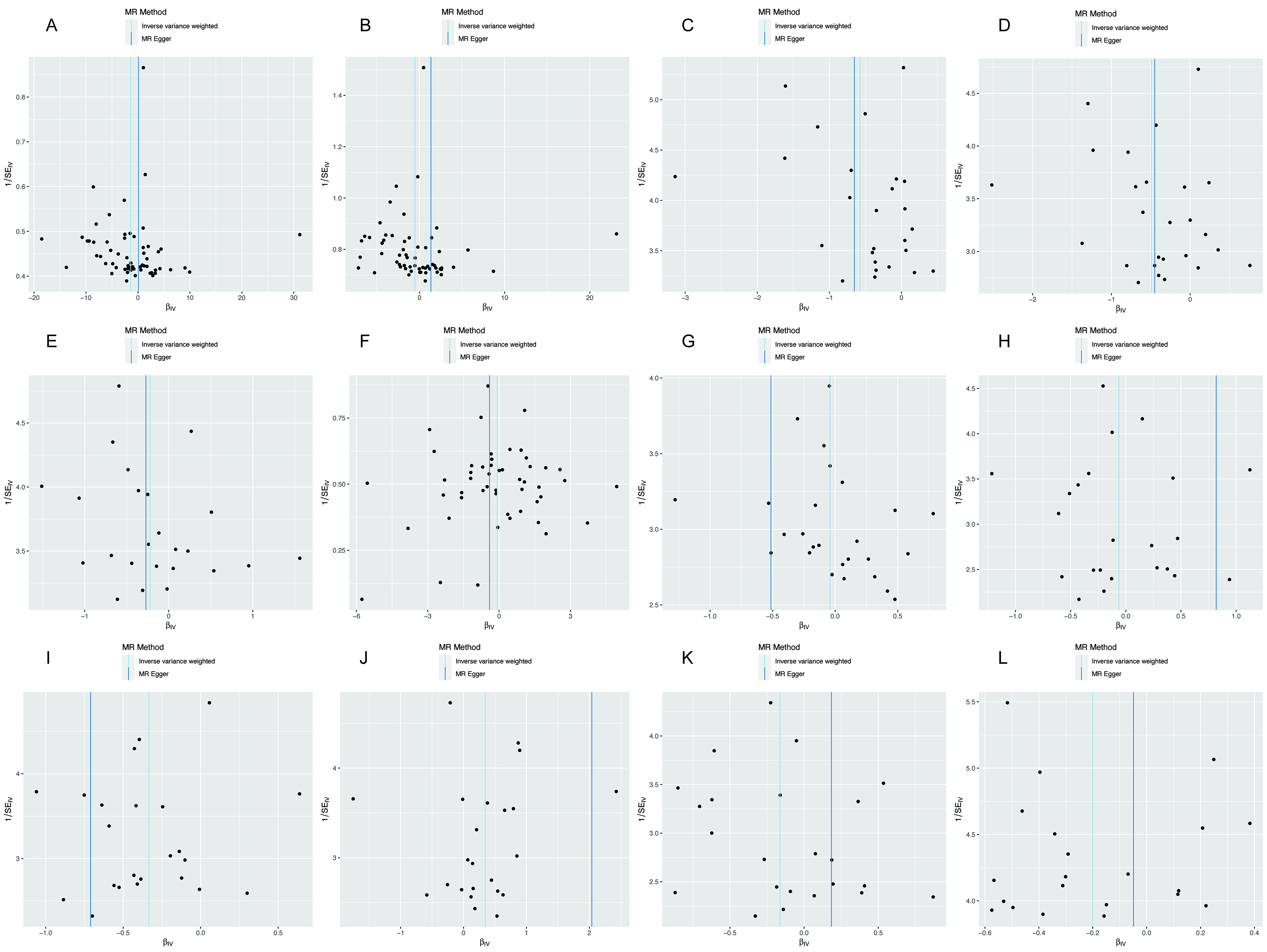

Supplement: Supplementary file 1 [file nutrients-14-02936-s001.zip › Supplementary Figure S8.tif]
